# Prognostication and prediction of spread through air spaces in non-small cell lung cancer

**DOI:** 10.1093/icvts/ivaf077

**Published:** 2025-03-27

**Authors:** Zamaan Hooda, Mara B Antonoff

**Affiliations:** Department of Thoracic and Cardiovascular Surgery, UT MD Anderson Cancer Center, Houston, TX, USA; Department of Thoracic and Cardiovascular Surgery, UT MD Anderson Cancer Center, Houston, TX, USA

**Keywords:** spread through air spaces, non-small-cell lung cancer, frozen section

Dear Editor,

We read with great interest the manuscript by Hashinokuchi *et al.* [1], investigating the outcomes of spread through air spaces (STAS) in patients undergoing resection for non-small cell lung cancer (NSCLC). Despite its recognition as a distinct invasion pattern, its characterization remains unclear [2,3]. Moreover, the impact of STAS extent, or distance from tumour margin to the STAS, on oncologic and survival outcomes is controversial [4]. This report provides much-needed information addressing this knowledge gap in the current literature.

In this study, NSCLC patients undergoing resection were stratified by the presence of STAS. Those with evidence of STAS were further categorized based on maximum spread distance (MSD) between the farthest STAS and tumour: limited STAS (MSD ≤1000 µm) and extended STAS (MSD >1000 µm). Importantly, individuals with extended STAS experienced worse recurrence-free survival (RFS) and overall survival (OS) compared to those with limited and negative STAS.

Although these findings are noteworthy, it is important to highlight that the definition of STAS distance used in this investigation differed somewhat from those previously used by other authors. Nevertheless, we commend the authors for contributing significant information regarding the prognostication of STAS. Given the poorer outcomes associated with STAS, its presence has been suggested as an indication for more extensive resections. Ultimately, enhancing intraoperative decision-making strategies is necessary to improve outcomes, and this was explored by Cao *et al.* [5]. In their work, the diagnostic accuracy of frozen section (FS) to identify STAS was evaluated in patients with cT1N0M0 NSCLC. These researchers found that STAS was found in 18.2% of FS samples and 26.4% of final pathology specimens. This finding demonstrated a sensitivity and specificity of 56.4% and 95.4%, respectively, of FAS identification of STAS. Evidently, further research is necessary to improve diagnostic and therapeutic modalities for NSCLC with STAS presence, which will translate into better outcomes for our patients.
